# Body Weight and Quality of Life Among Adolescents in Krakow

**DOI:** 10.34763/devperiodmed.20182202.160170

**Published:** 2018-06-30

**Authors:** Agnieszka Magiera, Elżbieta Sochacka-Tatara, Agata Sowa, Ryszard Jacek, Agnieszka Pac

**Affiliations:** 1 Katedra Epidemiologii i Medycyny Zapobiegawczej, Wydział Lekarski, Uniwersytet Jagielloński, Collegium Medicum, Kraków, Polska

**Keywords:** adolescents, quality of life, KIDSCREEN-27, underweight, overweight, obesity, młodzież, jakość życia, KIDSCREEN-27, niedowaga, nadwaga, otyłość

## Abstract

**Aim:**

The aim of this study was to assess the relationship between body weight and the quality of life among adolescents in Krakow, Poland.

**Material and methods:**

The study comprised 1291 pupils - 632 girls and 659 boys from 17 Krakow middle schools. Their quality of life (QoL) was assessed by means of the Polish version of the KIDSCREEN-27 questionnaire. Five dimensions of QoL were analyzed as low, average and high QoL according to Polish cut-off points. The body mass index (BMI) of the adolescents was classified as underweight, normal weight, or excessive weight according to Polish growth charts.

**Results:**

Low QoL was observed significantly more often in girls than in boys. Excessive weight among both girls and boys was found to be a risk factor for low QoL in the “Physical Well-being” dimension as compared to normal weight adolescents. Additionally, boys with excessive weight had a two-fold higher risk of low QoL in the “Social Support & Peers” dimension (OR=2.00; 95 %CI:1.14-3.50). Underweight was associated with higher risk of low QoL in the “Physical Well-being”, “Autonomy & Parents”, and “Social Support & Peers” dimensions, but only among boys.

**Conclusions:**

Both, underweight and excessive weight were associated with low QoL. Excessive weight in youth was linked mainly with lower physical well-being. Underweight was a predictor of low QoL only among boys in the dimensions related to physical health, as well as relations with family and peers.

## Streszczenie

**Cel:**

Celem pracy była ocena zależności pomiędzy masą ciała a jakością życia młodzieży w Krakowie.

**Materiał i metody:**

Do analizy włączono 1291 uczniów – 632 dziewczęta i 659 chłopców z 17 gimnazjów w Krakowie. Do oceny jakości życia wykorzystano polską wersję kwestionariusza do badania jakości życia dzieci i młodzieży KIDSCREEN-27. Analizowano 5 wymiarów jakości życia. Do oceny poszczególnych wymiarów jakości życia wykorzystano kategoryzację jakości życia na niską, przeciętną i wysoką, zgodnie z wynikami polskiej walidacji kwestionariusza KIDSCREEN-27. Masę ciała nastolatków sklasyfikowano jako: niedowaga, masa ciała w normie, nadmierna masa ciała na podstawie polskich siatek centylowych dla BMI.

**Wyniki:**

Niską jakość życia zaobserwowano istotnie częściej u dziewcząt niż u chłopców. Nadmierna masa ciała zarówno wśród dziewcząt, jak i chłopców była czynnikiem ryzyka wystąpienia niskiej jakości życia w wymiarze Zdrowie fizyczne w porównaniu do adolescentów z masą ciała w normie. Dodatkowo, chłopcy z nadmierną masą ciała mieli dwukrotnie wyższe ryzyko niskiej jakości życia w wymiarze Rówieśnicy i wsparcie społeczne (Isz = 2,00; 95% PU: 1.14-3.50). U chłopców niedowaga była powiązana z wyższym ryzykiem niskiej jakości życia w wymiarach: Zdrowie fizyczne, Niezależność i relacje z rodzicami oraz Rówieśnicy i wsparcie społeczne.

**Wnioski:**

Zarówno niedowaga, jak i nadmierna masa ciała były powiązane z wystąpieniem niskiej jakości życia. Nadmierna masa ciała u młodzieży była związana głównie z gorszym samopoczuciem fizycznym. Niedowaga była predyktorem niskiej jakości życia tylko wśród chłopców w wymiarach dotyczących zdrowia fizycznego jak również relacji z rodzicami i rówieśnikami.

## Background

The prevalence of improper body weight among children and adolescents has been systematically increasing since the end of the last century and is one of the key public health challenges in Poland and around the world. In Europe every year about 400 000 children and adolescents develop overweight and about 85 000 suffer from obesity [1]. In the Polish nationwide OLAF study that was conducted in the years 2007-2009 among adolescents aged 13-18, the frequency of overweight and obesity was 14.6-19.4% in girls and 10.3−13.0% in boys [2], whereas 13.7% of girls and 10.0% of boys aged 7-18 were classified as underweight [3]. Improper body weight may lead to many somatic effects, and an increased risk of many chronic conditions, inter alia: diabetes mellitus type 2, cardiovascular disease, unhealthy lipid profile, orthopedic problems, sleep apnea [4, 5, 6]. Moreover, being obese in adolescence is related to an increased risk of obesity in adulthood [7]. Underweight at a younger age may increase the risk of inhibited growth and development, resulting in short stature, lean body or low muscle mass, but can also cause an impaired immune system, anemia, disrupted hormone regulation, fertility issues and many other health problems [8]. Improper body weight among adolescents is not only related to physical health problems but also to psychological, emotional and psychosocial consequences. Adolescence is a significant stage of the human development when various changes of a physical, psychological and social nature occur and, at the same time, there is a high susceptibility to cultural ideals and beliefs shaped by the socio-cultural environment [9]. The physical and psychosocial changes in adolescence involve adjustment to the changing body size, functioning and personality that have to be accepted by young people [9]. Additionally, the contemporary model of a slender woman and a muscular man promoted by the mass media influence the social expectations of youth and may disturb the self-assessment of body weight and be a source of dissatisfaction with their bodies [9, 10]. Many studies indicate that a negative image of their own bodies affects the adolescents’ satisfaction with life; negative emotions, excessive criticism in relation to the size of the body lead to psychological problems and a deterioration of everyday active functioning [10, 11, 12].

The functional condition and well-being of a person can be described quantitatively, assessing the quality of life [13]. According to the definition of the World Health Organization, quality of life is *„perceiving their life positions in culture and value systems by the individuals and in relation to their goals, expectations, standards and interests”* [14]. For young people, well-being, satisfaction with themselves, a positive attitude towards themselves and other people, close relations with the family and peers and establishing new relations are significant influencing Qol factors [15]. Many studies indicate that excessive body weight is related to a deteriorating quality of life; adolescents with excessive body weight have a worse QoL in comparison with their peers with normal weight, especially as far as their physical wellbeing is concerned [5, 6, 7, 16, 17, 18, 19]. Fewer studies focus on the association between weight deficiency and a young person’s QoL, although the results indicate that weight deficiency might be connected with a lower self-esteem in comparison with normal body weight peers [19, 20].

The aim of the present study was to assess the relationship between improper body weight and the quality of life among junior high school students in Krakow.

## Material and methods

### Sample and data collection

A cross-sectional study was conducted among students from 17 junior high schools from different districts of Krakow between December 2013 and February 2015. Information about the study and its aims were given to the schoolchildren’s parents during periodical parent-teacher meetings. Only 70% of the parents have expressed their opinion and gave feedback about the child’s participation (either approval or refusal) in the study. Among those who responded, 14% refused their child’s participation in the study. The anonymous questionnaire, which took approximately 10-15 minutes to fill out, was administered in school classrooms and filled out individually by the adolescents in the presence of a research assistant. Data from 1465 adolescents were collected, which constituted 90.2% of those who agreed to participate. We excluded 174 questionnaires from this analysis because of missing data for the KIDSCREEN-27 questionnaire (n=69), weight or height (n=85) or age (n=8). Furthermore, we decided to exclude schoolchildren aged 12 (n=3) and 17 (n=9) because of small numbers and not being representative for this group of youth.

Finally, analysis was performed for 1291 adolescents, 632 (49.0%) girls and 659 (51.0%) boys. The sample comprised 391 adolescents from the 1st (30.3%), 462 from 2nd (35.8%) and 438 from 3rd years of junior high school (33.9%).

### Ethics

The study was reviewed and approved by the Jagiellonian University Bioethics Committee (approval number: KBET/143/B/2013). During the recruitment phase school principals of all the participating schools have given their approval for the study. Moreover, individual written informed consent was obtained from the parents of the participating students.

### Quality of life (QoL)

The quality of life was measured using the Polish version of the KIDSCREEN-27 questionnaire – child version [21]. The response range for each out of the 27 items is based on a 5-point Likert scale. The scale indicates the frequency of certain behaviors or feelings (1 = never to 5 = always) or the intensity of an attitude (1 = not at all to 5 = extremely). The time frame refers to the previous week [22, 23]. The final assessment covers 5 dimensions of QoL:

- Physical Well-Being (5 items) – teh level of physical activity, energy and fitness of the child/adolescent,
- Psychological Well-Being (7-items) – psychological well-being, such as positive emotions, satisfaction with life and the absence of loneliness and sadness,
- *Autonomy &Parents* (7 items) − bond with parents, the atmosphere at home and the extent to which the child/ adolescent feels loved and supported by the family,
- *Social Support &Peers* (4 items) − the quality of the child’s/adolescents’ social relations and interactions with friends and peers,
- School Environment (4 items) − feelings towards school and relationships with teachers [22, 23].

The Polish version of the KIDSCREEN-27 has shown both satisfactory validity and reliability to be used in the Polish population (Cronbach’s α for the subscale range from 0.791 to 0.878) [21].

Each participant’s QoL in each dimension (recoded to 0-100 scale) was categorized as low, average or high according to sex and age specific cut-point values for Polish adolescents, as described in the paper by Mazur et al. [21]. The detailed distribution of the quality of life in the studied population was described in detail elsewhere [24].

### Body weight status and other independent variables

The data about weight and height were self-reported by the adolescents. The body mass index (BMI) was calculated as a ratio of weight in kilograms (kg) and the square of height in meters (m^2^). Based on BMI growth charts from the OLAF study [25] − national up-to-date BMI cut-off values were applied to define a participant’s body weight status – the cut-offs used are presented in Table I. Based on the recommendation of Centers of Disease Control and Prevention, underweight was defined as BMI below the 5^th^ percentile, normal weight as ranging from the 5^th^ percentile to less than the 85^th^ percentile, and excessive weight (overweight and obesity together) was at or above the 85^th^ percentile of BMI distribution specific for sex and age [26].

**Table I:** The values of cut-off points for BMI to define underweight (less than the 5^th^ percentile) and excessive weight (over or at the 85^th^ percentile) based on Polish national references from the OLAF study [25]. Tabela I. Wartości punktów odcięcia dla BMI do zdefiniowania niedowagi (wartości poniżej 5 centyla) i nadmiernej masy ciała (wartości równe lub powyżej 85 centyla) w oparciu o polskie wytyczne opracowane w badaniu OLAF [25]

Age *Wiek*	Girls *Dziewczęta*		Boys *Chłopcy*	
	5th percentile *5 centyl*	85th percentile *85 centyl*	5th percentile *5 centyl*	85th percentile *85 centyl*
13	15.2	22.4	15.1	22.4
14	15.9	23.0	15.7	22.9
15	16.5	23.4	16.3	23.4
16	16.9	23.6	16.9	24.0

The other variables included in our analyses are: sex, age, regular physical activity and the presence of chronic conditions. “Regular physical activity” was assumed if the adolescent declared that he or she does sports regularly (at least once per week) and the “presence of chronic conditions” was based on the single question “Have you been diagnosed with any chronic disease/ condition?”.

### Statistical analyses

Descriptive statistics were presented as frequencies and percentages. To assess the risk of having a low Qol related to the body weight status among adolescents the logistic regression model was applied. Firstly, crude models were estimated, which included only the weight status group, while later the relationship between the weight status and different QoL domains were adjusted for age, regular physical activity and the presence of chronic conditions. In all the comparisons across the weight status groups, the reference group was the one with normal weight. The models were developed for boys and girls separately. All the analyses were performed using IBM SPSS Statistics 24, with statistical significance set at p<0.05.

## Results

The mean age of the participants was 14.6 years (SD=0.95) and their mean BMI was 19.99 kg/m^2^ ± 3.08 (19.69 kg/m^2^ in girls and 20.29 kg/m^2^ in boys; p<0.001). Using growth charts from the OLAF study [25] we found that 6.0% of the respondents were classified as underweight, 80.7% as normal weight, 13.3% as having excessive weight. The detailed sample characteristic by BMI categories was shown in table II. Underweight was more prevalent among girls compared to boys, whereas excessive weight occurred more frequently among boys. In addition, the prevalence of excessive weight decreased with age (p=0.003; Table II). In the group of adolescents who declared regular physical activity (at least once per week) we observed a higher prevalence of normal weight and a lower prevalence of both excessive weight and underweight as compared to those non-active.

**Table II:** Characteristics of the study population. Tabela II. Charakterystyka badanej grupy.

Variable *Zmienna*	Total *Ogółem* N=1291	Underweight *Niedowaga* N=77	Normal weight*Masa ciała w normie* N=1042	Excessive weight*Nadmierna masa ciała* N=172	Chi^2^ test;p-value *Chi^2^ test, wartość p*
	N	N(%)	N (%)	N (%)	
Sex					Chi2(2)=8.4;
*Płeć*					p=0.015
Girls/*Dziewczęta*	632	45 (7.1)	518 (82.0)	69 (10.9)	
Boys/*Chłopcy*	659	32 (4.9)	524 (79.5)	103 (15.6)	
Age					
*Wiek*					
13	184	3 (1.6)	150 (81.5)	31 (16.8)	Chi2(6)=20.1;
14	404	37 (9.2)	308 (76.2)	59 (14.6)	p=0.003
15	46	21 (4.5)	389 (83.3)	57 (12.2)	
16	236	16 (6.8)	195 (82.6)	25 (10.6)	
Regular physical activity*					
Regularna aktywność fizyczna*					Chi2(2)=6.8;
Yes	1197	68 (5.7)	975 (81.5)	154 (12.9)	p=0.033
No	91	9 (9.9)	64 (70.3)	18 (19.8)	
Chronic diseases**					
Choroby przewlekłe**					Chi2(2)=4.2;
Yes	207	17 (8.2)	157 (75.8)	33 (15.9)	p=0.124
No	1057	60 (5.7)	865 (81.8)	132 (12.5)	

*missing data (n= 3) not included/*braki danych (n=3) nie zostały włączone* **missing data (n=27) not included/*braki danych (n=27) nie zostały włączone*

The assessment of QoL was the lowest for the Physical Well-being and Psychological Well-being domains – more than 20% of the adolescents were classified as having low QoL in those domains (Table III). We observed significant discrepancies in the level of the QoL between girls and boys in four out of the five KIDSCREEN-27 dimensions: Physical Well-being, Psychological Well-being, Autonomy & Parents, School Environment, wherein girls assessed their QoL as low more frequently than boys (Table III).

**Table III:** Quality of life classification in KIDSCREEN-27 dimensions in girls and boys. Tabela III. Klasyfikacja jakości życia dziewcząt i chłopców w wymiarach KIDSCREEN-27.

		Total *Ogółem* N=1291	Girls*Dziewczęta*N=632	Boys*Chłopcy*N=659	Chi^2^ test;p-value *Chi^2^ test, wartość p*
		N (%)	N (%)	N (%)	
Assessment of QoL in every dimension of KIDSCREEN-27 *Ocena jakości życia w każdym wymiarze KIDSCREEN-27*	**Physical Well-being** ***Zdrowie fizyczne***				
	Low QoL *Niska jakość życia*	280 (21.7)	189 (29.9)	91 (13.8)	Chi2(2)=79.4;p<0.001
	Average QoL *Przeciętna jakość życia*	835 (64.7)	399 (63.1)	436 (66.2)	
	High QoL *Wysoka jakość życia*	176 (13.6)	44 (7.0)	132 (20.0)	
	**Psychological Well-being** ***Samopoczucie psychiczne***				
	Low QoL *Niska jakość życia*	333 (25.8)	235 (37.2)	98 (14.9)	Chi2(2)=84.1;p<0.001
	Average QoL *Przeciętna jakość życia*	788 (61.0)	329 (52.1)	459 (69.7)	
	High QoL *Wysoka jakość życia*	170 (13.2)	68 (10.8)	102 (15.5)	
	**Autonomy & Parents** ***Niezależność i relacje z rodzicami***				
	Low QoL *Niska jakość życia*	231 (17.9)	163 (25.8)	68 (10.3)	Chi2(2)=58.7; p<0.001
	*Average QoL* *Przeciętna jakość życia*	804 (62.3)	373 (59.0)	431 (65.4)	
	High QoL *Wysoka jakość życia*	256 (19.8)	96 (15.2)	160 (24.3)	
	**Social Support & Peers** ***Rówieśnicy i wsparcie społeczne***				
	Low QoL *Niska jakość życia*	198 (15.3)	106 (16.8)	92 (14.0)	Chi2(2)=5.3;p=0.069
	Average QoL *Przeciętna jakość życia*	807 (62.5)	375 (59.3)	432 (65.6)	
	High QoL *Wysoka jakość życia*	286 (22.2)	151 (23.9)	135 (20.5)	
	**School Environment** ***Środowisko szkolne***				
	Low QoL *Niska jakość życia*	243 (18.8)	149 (23.6)	94 (14.3)	Chi2(2)=19.4;p<0.001
	Average QoL *Przeciętna jakość życia*	878 (68.0)	411 (65.0)	467 (70.9)	
	High QoL *Wysoka jakość życia*	170 (13.2)	72 (11.4)	98 (14.9)	

The analysis of the association of weight status with QoL (Table IV) showed that Physical Well-being was significantly related to weight status – especially excessive weight was associated with a lower quality of life among both boys and girls. In addition, but only among boys, low QoL in the Social Support and Peers domain was related to improper weight (both underweight and excessive weight). The other three QoL domains were not dependent on the adolescents’ weight status (Table IV).

**Table IV:** Quality of life classification in KIDSCREEN-27 dimensions in relation to the body mass categories of girls and boys. Tabela IV. Klasyfikacja jakości życia dziewcząt i chłopców w wymiarach KIDSCREEN-27 w odniesieniu do kategorii masy ciała.

		Girls *Dziewczęta*			Boys *Chłopcy*		
		Underweight *Niedowaga* N=45	Normal weight *Masa ciała w normie* N=518	Excessive weight *Nadmierna masa ciała* N=69	Underweight *Niedowaga* N=32	Normal weight *Masa ciała w normie* N=524	Excessive weight *Nadmierna masa ciała* N=103
		N (%)	N (%)	N (%)	N (%)	N (%)	N (%)
**Assesment of QoL in every dimension of KIDSCREEN-27** ***Ocena jskości życia w każdym wymiarze KIDSCREEN-27***	**Physical Well-being** ***Zdrowie fizyczne***						
	*Low QoL*	13 (28.9)	142 (27.4)	34 (49.3)	10 (31.3)	54(10.3)	27 (26.2)
	*Average QoL*	26 (57.8)	339 (65.4)	34 (49.3)	15 (46.9)	350 (66.8)	71 (68.9)
	*High QoL*	6(13.3)	37 (7.1)	1 (1.4)	7 (21.9)	120 (22.9)	5(4.9)
	Chi2(4)=18.2; p=0.001				Chi2(4)=39.2; p=<0.001		
	**Psychological Well-being** ***Samopoczucie psychiczne***						
	Low QoL	14 (31.1)	196 (37.8)	25 (36.2)	6(18.8)	73 (13.9)	19 (18.4)
	Average QoL	26 (57.8)	267 (51.5)	36(52.2)	24 (75.0)	362 (69.1)	73 (70.9)
	High QoL	5 (11.1)	55 (10.6)	8 (11.6)	2 (6.3)	89 (17.0)	11 (10.7)
	Chi2(4)=0.9; p=0.926				Chi2(4)=5.8; p=0.218		
	***Autonomy & Parents*** ***Niezależność i relacje z rodzicami***						
	Low QoL	13 (28.9)	132 (25.5)	18(26.1)	7 (21.9)	53 (10.1)	8(7.8)
	Average QoL	22 (48.9)	309 (59.7)	42 (60.9)	19 (59.4)	344 (65.6)	68 (66.0)
	High QoL	10 (22.2)	77 (14.9)	9 (13.0)	6(18.8)	127 (24.2)	27 (26.2)
	Chi2(4)=2.8; p=0.599				Chi2(4)=5.6; p=0.234		
	**Social Support & Peers** ***Rówieśnicy i wsparcie społeczne***						
	Low QoL	6(13.3)	82 (15.8)	18(26.1)	9 (28.1)	61 (11.6)	22 (21.4)
	Average QoL	29 (64.4)	306 (59.1)	40 (58.0)	19 (59.4)	351 (67.0)	62 (60.2)
	High QoL	10 (22.2)	130 (25.1)	11 (15.9)	4(12.5)	112 (21.4)	19 (18.4)
	Chi2(4)=6.6; p=0.160				Chi2(4)=12.9; p=0.012		
	**School Environment** ***Środowisko szkolne***						
	Low QoL	16 (35.6)	117 (22.6)	16(23.2)	6(18.8)	74(14.1)	11 (13.4)
	Average QoL	26 (57.8)	339 (65.4)	46 (66.7)	20 (62.5)	371 (70.8)	58(70.7)
	High QoL	3 (6.7)	62 (12.0)	7 (10.1)	6(18.8)	79 (15.1)	13 (15.9)
	Chi2(4)=4.5; p=0.343				Chi2(4)=1.6; p=0.805		

The risk of having low QoL was assessed by means of the logistic regression model (Table V). In the crude models excessive weight, but not underweight, had an effect on the risk of low QoL in the Physical Well-being and Social Support & Peers domains among girls. However, after adjustment for possible confounders, a significant relationship for excessive body weight and risk of low QoL was found only in the domain of Physical Wellbeing (OR=2.48; 95%CI: 1.46-4.22). In boys excessive body weight influenced Physical Well-being (similarly to girls), as well as the Social Support & Parents domains. These results were significant also after adjustment for age, physical activity and the presence of chronic conditions. In addition, among boys, our study also showed the impact of underweight on the quality of life – the estimated effect was even stronger than among the adolescents assessed for excessive weight and was significant in the adjusted models. Underweight was related with a higher risk of low QoL in Physical Well-being (OR=3.41; 95%CI: 1.47-7.90), Social Support & Parents (OR=2.81; 95%CI: 1.22-6.49) and Psychological Well-being (OR=2.58; 95%CI: 1.05-6.33).

**Table V:** The odds ratio of low quality of life in KIDSCREEN-27 dimensions by body mass categories for girls and boys. Tabela V. Iloraz szans wystąpienia niskiej jakości życia w wymiarach KIDSCREEN-27 według kategorii masy ciała dziewcząt i chłopców.

	Girls *Dziewczęta*				Boys *Chłopcy*			
	Crude Model *Model surowy*		Adjusted* Model *Model standaryzowany*		Crude Model *Model surowy*		Adjusted* Model *Model standaryzowany*	
	OR	95% CI	OR	95% CI	OR	95% CI	OR	95% CI
***Physical well-being/Zdrowie fizyczne***								
Underweight *Niedowaga*	1.08	0.55-2.11	0.97	0.48-1.97	3.96	1.78-8.80	3.41	1.47-7.90
Normal weight *Masa ciała w normie*	1.00 (ref.)	-	1.00 (ref.)	-	1.00 (ref.)	-	1.00 (ref.)	-
Excessive weight *Nadmierna masa ciała*	2.57	1.55-4.28	2.48	1.46-4.22	3.09	1.84-5.21	2.60	1.47-4.60
***Psychological well-being/Samopoczucie psychiczne***								
Underweight *Niedowaga*	0.74	0.39-1.43	0.72	0.37-1.39	1.43	0.57-3.58	1.32	0.52-3.36
Normal weight *Masa ciała w normie*	1.00 (ref.)	-	1.00 (ref.)	-	1.00 (ref.)	-	1.00 (ref.)	-
*Excessive weight* *Nadmierna masa ciała*	0.93	0.55-1.57	0.89	0.53-1.52	1.40	0.80-2.44	1.44	0.81-2.54
***Autonomy & Parents/Niezależność i relacje z rodzicami***								
Underweight *Niedowaga*	1.19	0.61-2.33	1.18	0.60-2.31	2.49	1.03-6.03	2.58	1.05-6.33
Normal weight *Masa ciała w normie*	1.00 (ref.)	-	1.00 (ref.)	-	1.00 (ref.)	-	1.00 (ref.)	-
Excessive weight *Nadmierna masa ciała*	1.03	0.58-1.83	0.97	0.54-1.74	0.75	0.35-1.63	0.87	0.38-2.00
***Social Support & Peers/Rówieśnicy i wsparcie społeczne***								
Underweight *Niedowaga*	0.82	0.34-2.00	0.81	0.33-1.99	2.98	1.31-6.71	2.81	1.22-6.49
Normal weight *Masa ciała w normie*	1.00 (ref.)	-	1.00 (ref.)	-	1.00 (ref.)	-	1.00 (ref.)	-
Excessive weight *Nadmierna masa ciała*	1.88	1.04-3.78	1.74	0.95-3.17	2.06	1.20-3.54	2.00	1.14-3.50
***School Environment/Środowisko szkolne***								
Underweight *Niedowaga*	1.90	0.99-3.60	1.79	0.93-3.45	1.40	0.56-3.53	1.45	0.57-3.66
Normal weight *Masa ciała w normie*	1.00 (ref.)	-	1.00 (ref.)	-	1.00 (ref.)	-	1.00 (ref.)	-
Excessive weight *Nadmierna masa ciała*	1.04	0.57-1.88	0.95	0.51-1.76	0.96	0.52-1.80	1.00	0.53-1.91

*adjusted for age, regular physical activity, chronic conditions/*standaryzowany na wiek, uprawianie regularnej aktywności fizycznej*, wystąpienie chorób przewlekłych OR − odds ratio/*iloraz szans*; CI − confidence interval/*przedział ufności*

## Discussion

Our study, which was conducted on a large sample of Polish adolescents, indicated that girls reported a lower QoL than boys. This result is supported by many other studies worldwide [27, 28, 29]. Poorer QoL in girls might be explained by the biological and psychological differences between the sexes. The period of adolescence is a time of serious changes in a young man’s life, however girls experience all the changes in their bodies (the first menstrual period, increasing outer body fat) in a more negative way than boys [30, 31].

### The relationship between excessive body weight and the quality of life

Excessive body weight was a predictor of a young person’s low QoL in two KIDSCREEN-27 dimensions: Physical Well-being and Social Support & Peers. Both girls and boys with excessive body weight had a higher risk of low Physical Well-being compared to their peers with normal body weight. Interestingly, among girls this was the only dimension affected by body weight (after adjustment for confounders). We did not confirm the relationship of abnormal body mass with other QoL subscales. Many studies indicate the existence of a relationship between excessive body weight and a worse QoL in the dimension that refers to physical well-being and overweight and /or obesity seems to be related to a lower quality of life [7, 18, 19, 27]. Lower QoL among adolescents with excessive weight could be related to the negative health consequences that may be experienced by adolescents with overweight and obesity. Young people with excessive body weight experience difficulties in taking up physical activities, due to the lack of energy, vigor and physical strength. They usually avoid any sport, because they get tired faster and have worse motor activity [19, 32].

Among boys excessive body weight was also related with a higher risk of rating low in the Social Support & Peers dimension. Excessive weight among boys is a very frequent reason for teasing, mocking and rejection by peers. Adolescents with excess body weight often experience social exclusion and even bullying by peers [33, 34, 35]. Similar results, but in both sexes, were found in the studies conducted among American and Iranian adolescents, where young people with excessive body weight rated their social functioning as worse than their normal-weight peers [7, 16, 19]. On the other hand, observations in other populations do not confirm this relationship [17, 18]. It is worth mentioning that a higher risk of low QoL in the Social Support & Peers dimension among girls with excessive weight was observed only in the crude model, which took into consideration BMI exclusively. Many studies indicate that girls want to be perceived by others as desirable, attractive and to be accepted in their peer group [36]. However, we do not observe the relationship after the adjustment for age, regular physical activity and the presence of chronic diseases, thus this relation may have been due to the above factors, especially age.

We did not observe a significant relationship between excessive body weight and the other dimensions of QoL that were assessed, either among boys or girls. In our study we observed no significant relationship between excessive body weight and the young person’s mental health, which is in line with the studies of Cui et al. and Helseth et al [17, 32]. Researchers of the KIDSCREEN project also indicated that there was no significant relationship between excessive body weight and psychological wellbeing, which can be explained by the fact that this dimension mainly measures positive aspects, such as a sense of satisfaction and happiness [37]. However, some other authors indicated that obese adolescents suffer from depression, experience embarrassment and social degradation [7, 33]. The differences between studies can result from a different age distribution of the population analyzed by these studies. Swallen et al. showed that in the dimension of emotional functioning, overweight and obesity influenced the occurrence of depression and low self-assessment among younger obese adolescents, but not among older ones [16]. Moreover, we did not observe a relationship between excessive body weight and the QoL in the Autonomy & Parents dimension. This may be related to the fact that the parents of obese children are often obese themselves, so due to their own experience of being obese, this factor does not impair their relationships with their children. Moreover, it leads to their losing perception of excessive weight [38].

### The relationship between body weight deficiency and the quality of life

In our study, the relation between too low a body weight and low QoL was found only among boys. Underweight was a predictor of a low QoL score in the Physical Well-being, Autonomy & Parents and Social Support & Peers dimensions, to a greater extent than excessive weight. It is worth mentioning that there is little information on the relationship between body weight deficiency and the adolescent’s QoL in the literature available. In our study boys with underweight had an over threefold higher risk of low Physical Well-being than boys with normal body weight. This might be related to the fact that boys with underweight do not have well-built muscles, so they can have problems with taking up physical activities. The study of Swallen et al. showed that underweight adolescents were significantly more likely to have functional limitations [16]. When considering the Autonomy & Parents dimension among boys and its relation with underweight, one can suggest that a body weight deficiency may be associated with perceiving the adolescents’ health as worse by their parents, especially when compared with the ideal muscular stature of men subsequently having greater control over their bodies. It is also possible that underweight is related to the poor financial condition of the family, which means that the adolescents receive less money for their own expenses (the dimension Autonomy & Parents includes two questions referring to the financial resources of the respondents). However, a large study conducted on a nationally representative sample of Polish school-age children and adolescents does not confirm a significant relationship between family income and the risk of underweight [39]. The third dimension for which the relation between underweight and low QoL was observed among boys was Social Support & Peers. These results are in line with the study conducted among Chinese children [20]. This may be related to the fact that a boy with underweight may be easier to bully by his peers because of his body than a muscular boy, with an athletic body shape who can cause fear among peers [40]. The lack of this relation among girls can be partly explained by the observation of Olweus [34] who pointed out that physical bullying is more frequent among boys, and the victims are likely to be physically weaker than their peers or may simply perceive themselves as physically or mentally weaker than the perpetrator [34].

The presented study has raised questions why underweight is a more pronounced factor affecting QoL in Polish boys than girls, and why, unlike the results of many other studies, in Polish girls excess weight was significantly related only with physical limitations.

Perhaps this observation is an effect of mass-media created ideals: among boys, to be physically strong is synonymous of a high status among peers, while being skinny is an indication of weakness; among girls, there is no advantage in being physically superior, on the contrary there is great pressure on young girls to be slim. On the other hand, however, in girls with excess weight we have not observed a deterioration of QoL except in the area of the physical dimension. Maybe this is a consequence of the rising prevalence of excessive weight in the whole population, and thus greater acceptability of this phenomenon by young people.

### Limitations and strengths of the study

Some limitations of our study should be mentioned. Firstly, taking into account the fact that our research was of a cross-sectional nature, it did not enable the demonstration of the causality between improper body mass and QoL. Moreover, we did not measure the body weight and height of the young people studied, as the data concerning the body weight and height of the participants were obtained from the questionnaires filled in by the teenagers themselves.

The research also has some unique advantages. We tested the association between the body weight status and QoL on a large sample of adolescents from the general population. QoL was assessed by means of the KIDSCREEN-27 questionnaire, which was validated and used across many countries. In addition, both the classification of the body weight status and the QoL among the participants was made based on the Polish cut-off points specific for sex and age. It should be emphasized that our study observed a significant relationship between underweight and quality of life in boys, which is not a frequent finding. Most of the previous studies had paid attention mainly to the relationships between excessive body weight and different aspects of the youth’s QoL.

## Conclusions

In conclusion, our study reveals that both deviations from healthy body weight, underweight and excess weight, were associated with low QoL in different dimensions among the adolescents studied. Excess body weight was a predictor of low Physical Well-being in both boys and girls and moreover obtained a low rate in the Social Support & Peers dimension, but in boys only. Underweight was related to low QoL in three out of the five studied dimensions among boys: Physical Well-being, Autonomy & Parents and Social Support & Peers, but not among girls.
